# The COVID-19 Run on Medical Resources in Wuhan China: Causes, Consequences and Lessons

**DOI:** 10.3390/healthcare9101362

**Published:** 2021-10-13

**Authors:** Gaofeng Yin, Hanning Song, Jian Wang, Stephen Nicholas, Elizabeth Maitland

**Affiliations:** 1 Dong Fureng Institute of Economic and Social Development, Wuhan University, Wuhan 430072, China; yingaofeng367474@163.com (G.Y.); wangjian993@whu.edu.cn (J.W.); 2 School of Banking and Finance, University of International Business and Economics, Beijing 100029, China; 3 Australian National Institute of Management and Commerce, Sydney 2015, Australia; stephen.nicholas@newcastle.edu.au; 4 Newcastle Business School, University of Newcastle, Newcastle 2308, Australia; 5 School of Management, University of Liverpool, Liverpool L697ZH, UK; e.maitland@liverpool.ac.uk

**Keywords:** COVID-19 pandemic, medical resource runs, bank runs

## Abstract

The COVID-19 run on medical resources crashed Wuhan’s medical care system, a medical disaster duplicated in many countries facing the COVID-19 pandemic. In a novel approach to understanding the run on Wuhan’s medical resources, we draw from bank run theory to analyze the causes and consequences of the COVID-19 run on Wuhan’s medical resources and recommend policy changes and government actions to attenuate runs on medical resources in the future. Like bank runs, the cause of the COVID-19 medical resource run was rooted in China’s local medical resource context and a sudden realignment of expectations, reflecting shortages and misallocations of hospital resources (inadequate liquidity and portfolio composition); high level hospitals siphoning-off patients from lower level health providers (bank moral hazard and adverse selection problem); patients selecting high-level hospitals over lower-level health care (depositor moral hazard problem); inadequate government oversight and uncontrolled risky hospital behavior (inadequate bank regulatory control); biased medical insurance schemes (inadequate depositor insurance); and failure to provide medical resource reserves (failure as lender of last resort). From Wuhan’s COVID-19 run on medical resources, we recommend that control and reform by government enlarge medical resource supply, improve the capacity of primary medical care, ensure timely virus information, formulate principles for the allocation of medical resources that suit a country’s national conditions, optimize the medical insurance schemes and public health fund allocations and enhance the emergency support of medical resources.

## 1. Introduction

Wuhan was the epicenter of the COVID-19 pandemic, where panicked patients rushed Wuhan’s hospitals, creating a severe shortage of beds and over-taxing the medical staff [1]. While Wuhan’s medical services were the first to face a COVID-19 run on hospitals, many other countries, including the U.S., Germany, France, Peru, Brazil, Japan and Lebanon, all experienced runs on their medical services by COVID-19 patients. In the UK, COVID-19 patients overwhelmed hospitals, creating a critical care bed crises in the National Health System [2]. During the worst days of the pandemic in early 2020, more than a third of hospitals in the US were running short of doctors and nurses as COVID-19 patients swamped hospital services [3]. ICU beds, ventilators and healthcare workers in Italy and Switzerland were overcome by the rush on insufficient COVID-19 health care services [4,5]. Developing countries experienced a similar run on hospitals, where Covid-19 patient surges in Delhi and Jakarta caused acute shortages in hospital beds, medicines and lifesaving oxygen, with high death rates [6,7]. As far as we know, this is the first paper to use the prism of bank runs to analyze the run on hospitals during a pandemic. Wuhan’s health system is our case study: the first hospital system to experience the COVID-19 run; the first hospitals to respond to the COVID-19 run; and the first health system to provide lessons on the COVID-19 run on medical resources. Using bank runs to distil the causes, consequences and lessons of Wuhan’s COVID-19 hospital run, we provide a unique framework that can be applied to analyzing hospital runs generally. The first element of our hospital run framework was derived from Wuhan’s pre-pandemic experience with government hospital management and control, behavior of patients and hospitals and healthcare resourcing. The second element of the framework was Wuhan’s responses to the COVID-19 hospital run. While our hospital run framework is not time or space specific, its application needs to acknowledge each country’s specific hospital system and patient experiences. Our hospital run framework not only allows lessons to be drawn for Wuhan and China, but also allows a wider understand of how hospital systems globally cope when medical services are challenged by a pandemic. 

## 2. Materials and Methods

By drawing on a rich and extensive history of bank runs during financial crises, we provide a unique perspective to understand Wuhan’s run on medical resources during COVID-19. Bank panics are a sudden and destructive demand for deposit assets, stressing the bank’s solvency when large amounts of cash are withdrawn in a short period of time [8]. A run on a single bank can lead not only to the failure of that bank [9], but can cause the banking system to suffer a liquidity crisis, collapsing the entire financial system, such as the 1929–1933 Great Depression and the 2008 global financial crisis [10]. During a liquidity crisis, the banking system in the short run is unable to convert assets into cash to meet the cash withdrawals by depositors, resulting in banks failing [11]. This can stress a bank’s solvency, even when long-term assets exceed short-term liabilities.

Analogous to panic runs by bank depositors, the outbreak of the COVID-19 pandemic caused a “run” by patients on China’s medical resources, stressing both individual hospitals and the entire health system. Highly contagious, and before the development of the COVID-19 vaccine without a specific treatment, COVID-19 sparked a public panic for medical resources, where people snapped up and hoarded medical resources through over-treatment seeking behavior. Like a bank run, the COVID-19 panic left hospitals unable to “pay out” on the soaring demand for treatment. Like the withdrawal of depositor funds, the run on medical resources involved panic over-consumption of medical resources, equivalent to patient “withdrawal” of medical resources from hospitals. To provide new insights into how medical runs can be avoided and managed, we have two purposes: using the economic concepts of bank runs, first, to analyze the causes and consequences of the run on Wuhan’s health resources during the COVID-19 pandemic, and, second, to draw lessons for managing COVID-19 runs on medical resources in Wuhan, China and globally.

### Anatomy of Bank Runs: Imperfect Information

Bank runs reflect national bank system characteristics, where each bank run occurs in a specific historical context defined by time, place and national economic and social system, such as the U.S. bank failures in the 1930s Great Depression. For analytical purposes, bank run theory has synthesized the experience of different bank runs at different times and different countries into a broad theoretical framework. Given the local context, the similarities across bank runs have been theorized as a sudden realignment of expectations, triggered by asymmetries in information between depositors and banks [12,13,14,15], giving rise to moral hazard and adverse selection problems, which explain the crash of banking systems [16,17]. We apply bank run analytical tools, especially problems arising from imperfect information, to understand the causes and consequences of the run on medical resources in Wuhan during COVID-19.

Bank runs are based on asymmetric, or imperfect, information, where banks and depositors have different information, and banks and regulators have better information [12]. Asymmetric information gives rise to different behavior by each actor that causes banks and the banking system to fail [13]. Depositors display moral hazard by investing in risky banks and risky asset portfolios, comprising stocks, bonds, mutual funds, derivatives, cash and cash equivalents [18,19]. For banks, when individual depositor’s bank deposits are insured by the country’s central bank, asymmetric information causes both moral hazard and adverse selection behavior that leads to the failure of the banking system. When banks have better information than depositors and regulators, banks have a motive to engage in risky practices, including unsafe lending and biased portfolio structures, which are hard for depositors and regulators to identify—the so-called moral hazard problem [20]. The moral hazard problem is a class of behaviors where actors undertake risky actions, but do not share the full cost of such behaviors [21]. 

In bank runs, bank adverse selection rests on a two-sided asymmetric information problem between depositors-banks and banks-regulator. Depositors are poorly informed about the bank’s long-term asset quality, or solvency, and banks cannot observe the true short-term liquidity needs of their depositors [14]. Given asymmetric information, banks engage in adverse selection, when the quality of borrowers declines, with the bank making risky loans to poor quality clients, but depositors can only imperfectly assess banks’ risky lending [22]. Since their deposits are insured, depositors have both a weak incentive to monitor banks and asymmetric information means depositors also have only limited ability to monitor banks, which allows banks’ adverse selection and moral hazard behavior to go unchecked by depositors [23]. 

Imperfect information also helps explain the run on banks. Depositors receive “noisy” information or unsubstantiated rumors about the health, operation, portfolio structure and long-term asset status of a bank, which leads depositors to rush to withdraw their deposits from ‘risky’ banks. The ‘risky’ bank then fails [14]. When one ‘risky’ bank fails, depositors in other solvent banks are also unable to assess their own banks’ fundamentals, setting the scene for system-wide banking failures. Fearful of losing their deposits, depositors withdraw their funds from safe and unsafe banks, as part of the contagion process, or a depositor behavioral response to changing expectations, which crashes the banking system [24,25].

Anti-bank run measures focus on control, both to attenuate the chance of a run and to attenuate the run itself. To attenuate the chance of a run, bank regulators mainly focus on oversight and on implementing a deposit insurance system, which guarantees deposited funds up to some predetermined ceiling [26]. The deposit insurance scheme means that there is no need to withdraw funds from failing banks below the deposit insurance ceiling since these funds are guaranteed by an external independent insurer who cannot go broke, usually the country’s central bank. However, such a central bank guarantee to depositors can encourage bank moral hazard behavior and adverse selection, where banks make risky loans, have insufficient long-term reserves and create unbalanced portfolios [27]. To address this moral hazard and adverse selection problem exacerbated by the deposit insurance scheme, the central bank as guarantor exercises oversight, regulating the bank’s lending, liquidity, long-term asset portfolio and reserves. During a bank run, the lender of last resort system allows the central bank to attenuate the impact of depositor runs and maintain financial stability through loan guarantees to individual banks, or the whole banking system [28]. The role of lender of last resort by the central bank means that when commercial banks or other financial institutions are fundamentally solvent, but have temporary liquidity difficulties and are unable to obtain financing from other banks or financial markets, they can seek short-term assets from the central bank through rediscounting or refinancing. Below, we apply the bank run framework to explain the causes and consequences of the COVID-19 run on Wuhan’s health system and offers lessons for preventing and controlling pandemic runs on hospitals.

## 3. Results

### 3.1. Context, Moral Hazard and Adverse Selection: China’s Health Care System before Covid-19

Like the banking system, runs on medical resources reflect the context of China’s hospital system. In China, health care is delivered via a three-tiered system, which provide a range of services from preventative health care to advanced medical treatment. Providing preventive and primary care services, primary care facilities comprise village clinics, township hospitals and community health centers, usually with 20–99 beds. With 100–499 beds, tier two county and district secondary hospitals provide basic specialty care and inpatient services. With over 500 beds, municipal hospitals are large-scale city-based tertiary hospitals, providing complex healthcare and advanced medical training and research [29]. Since the medical reform in 2009, China has built a universal healthcare system including the world’s largest basic medical insurance network covering the entire Chinese population [30]. In 2020, 1.36 billion Chinese people had basic insurance, which covered more than 95% of the population [31,32,33]. China has three major social health insurance schemes: the Urban Employee Basic Medical Insurance (UEBMI), covering employed workers; the Urban Resident Basic Medical Insurance (URBMI), covering children, students and other non-working urban residents [34]; and the New Rural Co-operative Medical Scheme (NRCMS), covering the rural poor [35]. There is no official open data about the population coverage of private health insurance, but a 2020 survey estimated a coverage rate around 4% in 2004–2006 and about 12% in 2013. The study argued that the contribution of private health insurance to extending China’s universal healthcare system was limited [36]. Local-provincial-central governments each play different financing and control roles, with the central government formulating major guidelines and policies, and subsidizing the service fee structure of public hospitals through financial allocations, with provincial and local governments financing hospitals, coordinating the allocation of medical resources and constructing medical and health service systems in their respective jurisdictions [37,38].

Patient choice is a key contextual factor in China’s pre-pandemic health care system. Characterized by imperfect information, patient preferences were biased towards tertiary hospitals, reinforced by the small price gap between service costs at the three different types of hospitals: the outpatient registration and consultation fee for an attending physician was RMB 3.5 in primary, RMB 4 in secondary and RMB 5 in tertiary hospitals [39,40,41]. The result was the over-use of higher-level hospitals and under-use of primary health care facilities.

**Figure 1:**
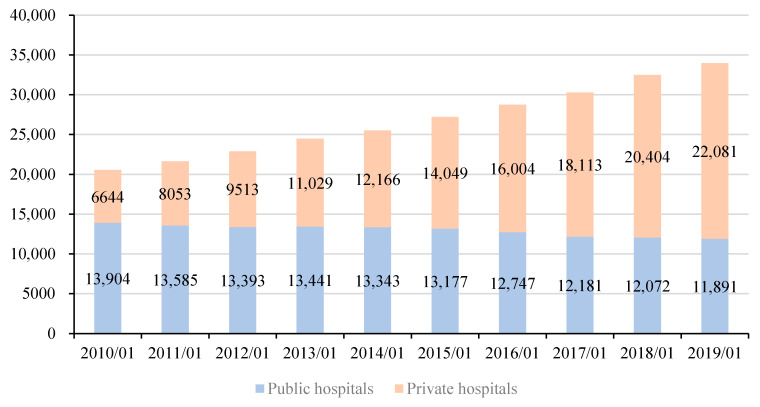
Number of public hospitals and private hospitals nationwide from 2010 to the end of November 2019. Note: The first case of new coronary pneumonia in China was discovered in Wuhan on 8 December 2019. In order to reflect the number of public and private hospitals in China before the outbreak, the latest phase of data uses the data at the end of November 2019. In order to reflect the monthly year-on-year changes, the data of other years are also used at the end of November. Data: National Health Commission of the PRC.

Comparable to reserve requirements that ensure bank liquidity and solvency, the viability and stability of China’s health care system rests on minimum resource levels. Before the COVID-19 outbreak, China and Wuhan’s medical resources were barely sufficient to cover normal demand. Inadequate long-run medical resources were due to inadequate investment in hospitals and the misallocation of existing health care resources between hospital tiers and urban-rural locations, reflecting a failure in hospital regulation. Figure 1 shows that the increase in the supply of hospitals in China relied on the increase in the number of private hospitals, with the number of public hospitals declining. At the end of 2017, China had 4.34 hospital beds per 1000 people, which was significantly less than Japan (13.05 beds per 1000 people), South Korea (12.27 beds per 1000 people), Russia (8.05 beds per 1000 people) and Germany (8 beds per 1000 people) [42]. Between 1978 and 2018, the growth in the number of hospital beds in China increased 3.5 times, significantly slower than the growth of China’s economy at nearly 250 times in the same period [43]. Shortages of hospitals occurred alongside a shortage of medical workers, especially nurses. As shown in Table 1 for the 2007–2016 period, China averaged 1.8 doctors and 2.3 nurses and midwives per 1000 people, with a ratio of 1 doctor to 1.28 nurses that was significantly lower than that of developed countries. While China had increased licensed physicians to 2.16 per 1000 people and registered nurses to 2.94 per 1000 people by 2018, the ratio of medical staff to nurses was only 1:1.14, with primary health facilities, especially in rural areas, especially under-resourced relative to tier 2 and tertiary hospitals [44].

**Figure 2:**
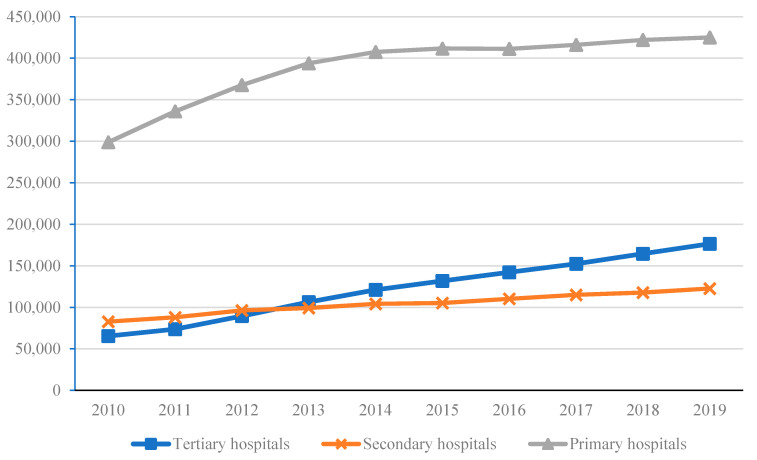
Annual cumulative total number of patients in medical institutions at all levels (10,000 people). Data: National Health Commission of the PRC.

Like banks, pre-COVID-19 tertiary hospitals in China displayed moral hazard behavior when imperfect information meant patients could not accurately assess the stability and long-run resource quality of hospitals in the tiered hospital system. Relying on their top-grade healthcare staff and equipment, profit-oriented tertiary hospitals “siphoned-off” patients from smaller hospitals and primary health centers, which exacerbated the shortages in medical resources through the over-use of medical resources at higher-level hospitals, and under-use of resources at lower-level hospitals [45,46]. In addition, the health insurance schemes made it easier for tertiary hospitals to “siphon-off” patients. This is because the health insurance schemes have improved patients’ ability to pay for expensive health services, and more patients were willing to enjoy tertiary hospitals rather than primary care facilities [47]. The existing Chinese public insurance policies also exacerbated moral hazard by patients. After China’s health care reform, higher coverage led to an increase in total health care consumption [48]. The increase in China’s health care medical expenses was partly due to “unnecessary” utilization of health care services by insured patients, who choose higher level hospital rather than primary health care treatment [49]. Figure 2 shows that the number of patients in tertiary hospitals rapidly increased since 2010, while the growth rate of patients in secondary hospitals and primary care facilities stagnated. 

Siphoning from lower-level hospitals created an excess medical services demand reflected in a “difficult to see a doctor” problem at tertiary hospitals, making tertiary hospitals vulnerable to runs [50]. One solution to over-use was rationing access through the 2009 hospital doctor reservation registration system [51]. However, the absence of default costs to cancel a hospital appointment gave rise to a second moral hazard problem, this time by patients. Appointments made, but not kept, imposed on hospitals the costs of unused doctor time and imposed on other patients the cost of being unable see an “unused” doctor, but imposed no costs on patients cancelling appointments. 

Bank adverse selection occurs when the quality of borrower declines. Hospital adverse selection occurs when patients could be adequately or better treated at primary hospitals but were induced to seek medical care at higher-level hospitals, resulting in the over-use (and misuse) of secondary and tertiary hospital resources. Health insurance schemes of China highlighted the “inverted pyramids” phenomenon. The need to be hospitalized is widely considered to be only for serious diseases by Chinese people, and they insist that only high-level hospitals provide good treatment. The reimbursement rate for UEBMI was higher than URBMI or NRCMS, leading patients to choose a higher-level hospital over primary health facilities [52]. Research in Fuxin city showed that UEBMI enrollees, who can get a higher compensation ratio, were more willing to go to tertiary hospitals than URBMI enrollees, who receive a lower compensation ratio [53]. Health insurance results in the greater use of hospital resources, but differential health insurance compensation leads to both increased and over-use of higher-level hospitals compared to primary hospitals. Over-use caused shortages in higher-level hospital resources; denied access to higher-level hospitals by patients requiring expert medical staff and sophisticated medical technology; and underutilized primary health facilities. Over-use exacerbated the “difficult to see a doctor” and “book a doctor” reservation system problems, and misallocated hospital resources [54]. 

**Figure 3:**
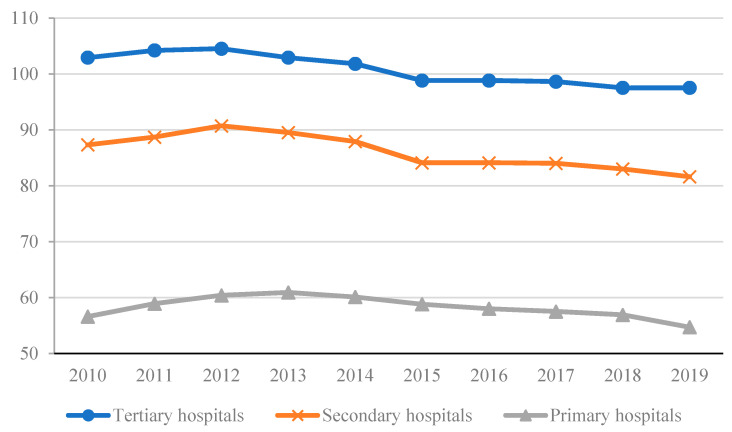
Average annual bed utilization rate of hospitals at all levels, township health centers and community health service centers. Data: National Health Commission of the PRC.

Moral hazard bank behavior leads to unbalanced long-run bank asset portfolios. Asymmetric information explained a failure in hospital regulation, which allowed medical resources to be unbalanced. The misallocation of medical resources, with most advanced medical technology, equipment and expert medical teams concentrated in tertiary hospitals, created long-term unbalanced medical resource portfolios across the primary care, secondary and tertiary systems and exacerbated the shortages in medical resources, compounding any run on the hospital system. The utilization rate of hospital beds in Figure 3 shows that tertiary hospitals operated at full capacity, significantly higher than the operating capacity of secondary and primary care facilities. During the pandemic, hospitals expanded the number of beds, built temporary hospitals and tried to allocate patients to the appropriate hospital tier for treatment [55]. However, the shortage of medical experts, outdated equipment and low diagnosis and treatment technology meant secondary and primary hospitals did not have the capacity to absorb the significantly greater additional health care demand during the pandemic. As a result, measures to force patients to visit lower health tiers during the pandemic were constrained [56,57,58].

The second aspect of the misallocation of the portfolio of medical resources that compounds shortages in medical resources was the uneven regional distribution of medical resources. In China, tertiary hospitals can be further divided into 3A-3C categories based on the quality of medical resources [59,60]. About 20% of all tertiary 3A hospitals at the end of 2018 were located in Beijing (55 3A hospitals), Guangzhou (38 3A hospitals), Shanghai (32 3A hospitals), Tianjin (31 3A hospitals), Chongqing (31 3A hospitals) and Xi’an (27 3A hospitals), with a smaller number in Wuhan (27 3A hospitals) and Chengdu (27 3A hospitals). The number of 3A hospitals in large cities was significantly larger than 3A hospitals in small and medium-sized cities and towns. Even more dramatic was that 4.67 million health personnel were located in urban hospitals at the end of 2018, but only 2.7 million health personnel were located in rural hospitals. The long-run structural and regional misallocation of medical resources, which hospital regulators failed to correct, facilitated the COVID-19 run on Wuhan hospitals.

### 3.2. The Anatomy of a Pandemic Run on Medical Resources in Wuhan

**Figure 4:**
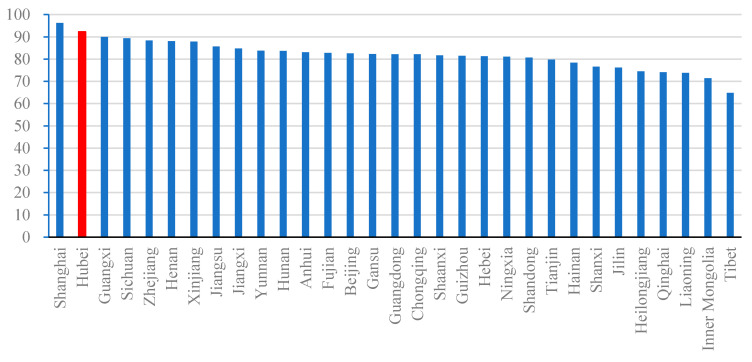
Utilization rate of hospital beds in 31 provinces in Mainland China in 2019 (%). Note: The bar in red shows the condition of Hubei, and the other bars are in blue. Data: National Health Commission of the PRC.

Moral hazard and adverse selection problems due to asymmetric information between hospitals, patients and regulators compounded the stretched and unbalanced medical resource portfolios, reinforced risky hospital behavior and led to regulatory failures, setting the stage for the run on Wuhan’s hospitals during the COVID-19 pandemic [61,62]. At the center of Wuhan’s COVID-19 breakout was an over-stretched and unbalanced hospital system, with Hubei province, and its capital, Wuhan, at 95% hospital bed utilization rate, which was significantly higher than the national average of about 87% over the 2010–2018 period [63]. As shown in Figure 4, the 2019 hospital bed utilization rate in Hubei Province ranked second in China; an average of 0.64 beds per 10,000 people, far below the national standard of 1.3 beds per 10,000 people; and before the outbreak of COVID-19, Wuhan had only two large over 900 bed specialized infectious disease hospitals. The size of infectious diseases department in Wuhan hospitals was also limited, and emergency infection wards were also in short supply. Wuhan hospitals had insufficient resources to deal with the COVID-19 outbreak [64].

Bank runs reveal the failure of bank regulators to provide oversight, including ensuring adequate long-run and short-run reserves, balanced portfolios and policies that attenuate moral hazard bank behavior [28]. In China, government oversight failed to address the hospital portfolio imbalances, with the misallocation of medical resources across hospital level and geographical space [65]. The biggest government regulatory failure was highlighted by the shortages in medical resources, including reserve requirements, with hospitals issuing appeals for material support from non-governmental organizations during the pandemic [66]. During the run on hospitals, hospital oversight remained inadequate, with a failure to coordination and control donated supplies, resulting in serious backlogs in the distribution of donated frontline materials and the misallocation of emergency medical supplies [64]. During the emergency pandemic response, the shortage and supply issues reflected broader omissions in emergency management by relevant local government departments, including emergency manpower shortages and inadequate interdepartmental coordination [67,68]. 

Bank runs occur due to information asymmetries, when depositors react to information, true or false, that bank resources are insufficient to meet depositor demand [12,13,14,15]. During the COVID-19 outbreak, the relevant government departments failed in the timeliness of accurate information about the causes, transmission and risks of the pandemic. Imperfect and incomplete COVID-19 information led Wuhan’s patients to rush the hospital system, especially high-level hospitals [62]. Comparable to depositor bank withdrawals, patients who believed themselves to be in danger of COVID-19, “withdrew” resources from hospitals by seeking medical care. Long lines of patients queuing for treatment led to contagion, with other Wuhan residents rushing for treatment before medical resources were exhausted [69]. A lack of government sourced COVID-19 information was a key element in the run on Wuhan’s medical resources [70].

While the Chinese government invested in a direct online reporting system for infectious diseases after the SARS epidemic, the direct reporting function of COVID-19 was not activated until 24 January 2020, which denied the government and the public accurate early information on the spread of the virus [71]. During the first stage of the pandemic, limited official disclosure of information raised the level of information uncertainty and lead people to seek, and believe, inaccurate gossip and rumor [72]. The lack of accurate information from the mainstream media brings about the “bad news syndrome”, where negative and untrue news floods online media [73]. Accurate and inaccurate media reports on COVID-19 cases, deaths, mass quarantines and public transport closures contributed to the uncertainty and public anxiety over the facts of the pandemic, exacerbating the overcrowding of hospitals. In addition, the COVID-19 outbreak caused anxiety disorders, related to the concerns of being infected, delayed return to work, stagnation of social activities and worries about the uncertain future [74].

## 4. Discussions: Lessons from Wuhan: How to Prevent Runs on Medical Resources 

### 4.1. Hospital Shortages and Bank Reserves

Comparable to a lack of liquidity, where banks have insufficient reserves to cover depositor withdrawals, the COVID-19 hospital run occurred against the context of a pre-pandemic shortage of medical resources. Bank deposit reserve regulations attenuate the probability of runs [75]. In the medical field, the government needs to specify a reasonable long-run reserve of medical resources, based on a combination of regular assessments and irregular spot checks. National and local government cooperation needs to assess whether medical resources match local needs, and whether the distribution of medical resources between regions and hospital levels are balanced. Implemented during the pandemic, the government should expand telemedicine to address medical resource shortages, overcome geographical imbalances and provide flexibility in the distribution of the nation’s medical resources [76,77].

### 4.2. Attenuating Moral Hazard and Adverse Selection Problems in Health Services

The moral hazard and adverse selection problem inflict both banks and hospitals, which lead to risky practices. Moral hazard behavior by hospitals resulted in higher-level hospitals siphoning-off low priority patients from primary and secondary hospitals; hospital adverse selection involved hospitals servicing patients better treated at lower-level medical facilities; and patients displayed moral hazard behavior by attending higher-level hospitals rather than equally suitable primary health care facilities. Enhanced oversight of hospital behavior, including holding appropriate reserves of medical resources, enhancing the primary health facility gatekeeper role and addressing the misallocation of medical resources, especially over-investment in tertiary hospitals and over-investment in urban health services, requires government regulation. ‘Big data’ and health information technology would provide regulators with better information to oversee hospital behavior and provide hospitals and patients enhanced information to attenuate patient moral hazard and hospital moral hazard and adverse selection behavior [78]. Another area requiring government regulation is pricing access to health facility, including co-payments and insurance reimbursements, to encourage more optimal use of lower tier hospitals, shifting patient preferences towards primary and secondary hospitals. For example, changing the differential in outpatient registration and consultation fee for an attending physician from the existing RMB 3.5 in primary, RMB 4 in secondary and RMB 5 in tertiary hospitals would reduce frivolous demands and condition patients to self-regulate access to the tiered hospital system.

### 4.3. Addressing the Misallocated Health Resource Portfolios 

In order to avoid the bank assets and liabilities mismatch, bank regulators require liquidity buffers and long-term diversified asset portfolios to enable credit institutions to withstand a liquidity stress for short periods of time [79]. Hospital resources were misallocated between levels of hospitals and regionally, where resource buffers were inadequate to allow the shift of resources between different level of hospitals and between urban and rural hospitals. Borrowing from the Basel risk weighting approach, government might weight healthcare factors, such as the complexity of treatment, need for specialist clinical resource, immediacy of treatment needs and patient co-morbidities, to more effectively allocate health resources and to signal to the public appropriate use of tiered hospital resources [80]. To change the structure of China’s medical resource portfolio, lower tier hospitals require incentives to attract qualified medical staff as well as material resources. There should be a strengthening of the capacity of primary medical care, including the establishment of rapid diagnosis and treatment systems that spans community health service centers, primary disease control institutions, and general hospitals. As part of hospital structural reform, transparent principles for the allocation of medical resource portfolios would address resource inadequacies at disadvantaged medical institutions [81] and address the public’s concerns about the quality of different hospital tiers. Following the bank run prevention model, the government should optimize the hospital financial compensation model, implement a reformed compensation system, and appropriately fund small and medium size medical institutions with weak self-support capabilities [82].

### 4.4. Reforming the Medical Insurance System

The introduction and improvement of the bank deposit insurance system has made depositors more trustworthy of small and medium-sized banks and reduced the liquidity pressure on large banks, thereby improving the overall resource allocation capacity of the banking system. The bank deposit insurance system reinforces the banking system’s ability to withstand panic runs. Hospital use and resource allocations were influenced by access to medical insurance. To address the problems of uneven, insufficient and incomplete coverage of medical insurance, further reforms to the medical insurance schemes and public health fund allocations are required. The medical insurance system reforms should align the benefit register across the main state insurance schemes, to allow insurance scheme portability regionally and to level-up local and provincial insurance funding. Reformed medical insurance should connect medical services between regions and urban-rural locations, which would help address structural and regional resource misallocations. Also, the regulation of insurance rebates would incentivize patients to use low-cost primary hospitals rather than high-cost tertiary hospitals by adjusting the out-of-pocket expenses.

### 4.5. Enhancing the Government as “Provider of Last Resort” for Medical Resources

To reduce runs and sustain the banking system, central banks act as “lender of last resort”. Government is seen as “provider of last resort”, guaranteeing medical resources during pandemics. During COVID-19, the central, provincial and local governments’ guarantee function for local emergency management was inadequate. As the provider of last resort, the central government must guarantee local hospitals adequate short-term resources during major public health emergencies and undertake the management of medical resource collection and deployment. While the Chinese government built new COVID-19 hospitals (adding to care capacity), repurposed existing elective beds and expanded telemedicine, long-term plans for managing pandemics and other health emergencies should be designed. Expanding immediate treatment capacity through COVID-19 hospitals turned out to be a short-term response to long-term resource shortages and misallocations [83]. Building new hospitals requires a long-term resource commitment and resource reallocation, including changing the structure of the tiered hospital system and resource allocation between urban and rural areas. Such long-term resource adequacy changes also require better inter-governmental coordination than existed in the pre-COVID-19 period. Second, the government needs to strengthen medical emergency drills. Similar to bank system “stress tests” by bank regulators, the government should run stress tests to gain expertise in building emergency hospitals, recruiting additional non-local medical staff and deploying central reserves to fund and manage local emergency measures, including the procurement of medical supplies. One recommendation is that the government should establish a big data platform to facilitate the supply of emergency medical supplies [84].

## 5. Conclusions

There are similarities in the causes, consequences and countermeasures between bank runs and medical resource runs. Based on the experience of the COVID-19 run on Wuhan’s medical resources and the anatomy of bank runs, we recommend the Chinese government take control measures to enlarge and nationally coordinate medical resource supply; improve the capacity and use of primary medical care; formulate principles for the allocation of medical resource portfolios between hospital levels and geographically; further reform medical insurance schemes and public health fund allocations to optimize hospital use; and enhance emergency support to address medical resource shortages. We finally suggest that Wuhan’s experience was not untypical of medical resource runs in developed and other developing countries during the COVID-19 pandemic. China’s experiences and lessons from the COVID-19 pandemic provide useful lessons for other countries.

## Figures and Tables

**Table 1:** Healthcare staffing in BRICS and developed economies, 2007–2016.

Country	Doctors Per 1000 Population	Nurses and Midwives Per 1000 Population	Ratio of Doctors to Nurses
China	1.8	2.3	1:1.28
Russia	4	8.7	1:2.18
Brazil	1.9	7.4	1:3.89
South Africa	0.8	5.2	1:6.50
India	0.8	2.1	1:2.63
Japan	2.4	11.2	1:4.67
UK	2.8	8.4	1:3.00
France	3.2	10.6	1:3.31
Germany	4.2	13.8	1:3.29
Norway	4.4	17.8	1:4.05
Switzerland	4.2	18.2	1:4.33

The ratio of doctors to nurses is calculated by comparing physicians, nurses and midwives per 1000 population. Data: 2019 China Health Statistics Yearbook, National Health Commission of the PRC.

## Data Availability

Publicly available datasets were analyzed in this study. This data can be found here: http://www.nhc.gov.cn/ (accessed on 13 October 2021).
